# Evaluation of an alternative biotreatment for the extraction of harmful iron and sulfur species from waterlogged wood

**DOI:** 10.1140/epjp/s13360-021-01908-9

**Published:** 2021-09-14

**Authors:** Mathilde Monachon, Magdalena Albelda-Berenguer, Tiziana Lombardo, Emilie Cornet, Friederike Moll-Dau, Janet Schramm, Katharina Schmidt-Ott, Edith Joseph

**Affiliations:** 1 Laboratory of Technologies for Heritage Materials, University of Neuchâtel, 2000 Neuchâtel, Switzerland; 2 Swiss National Museum, 8910 Affoltern am Albis, Zürich, Switzerland; 3 Haute Ecole Arc Conservation-Restauration, HES-SO University of Applied Sciences and Arts Western Switzerland, 2000 Neuchâtel, Switzerland; 4 Archaeological Service of Canton Bern, 3001 Bern, Switzerland

## Abstract

An innovative bioextraction method was tested and compared to common chemical extraction for the preservation of waterlogged archeological wood (WAW) artifacts. During burial, WAW artifacts accumulate iron and sulfur species forming iron sulfides. These compounds are harmless in the burial environment, where the oxygen content is low. But upon excavation, the WAW undergoes the oxidation of these compounds, and thus, irreversible physical and chemical damages occur. Fresh and archeological oak and pine samples were selected as representative species of WAW artifacts. Fresh samples were previously artificially contaminated to ascertain the presence of iron and sulfur. *Thiobacillus denitrificans* and natural iron chelators, called siderophores, were investigated to extract iron and sulfur as a 2-step biological treatment (BT) and compared to sodium persulfate–EDTA as chemical treatment (CT). Consolidation and freeze-drying were performed on the samples after BT and CT as traditional conservation protocols. BT and CT efficiency was evaluated through Raman, inductively coupled plasma–optical emission (ICP-OES), and Fourier transformed infrared (FTIR) spectroscopies. Raman and ICP showed that most of the iron and sulfur was extracted after BT, while some sulfur species remained present on CT samples. None of the extraction methods resulted in a degradation of the wood, as ascertained by FTIR analyses. Yet, all samples presented visual modifications after conservation. Pine samples treated with BT illustrated the oxidation of the species. Present principal component analysis (PCA) and analysis of variance (ANOVA) which were selected as statistical approaches and validated BT as a promising alternative extraction method, with encouraging extraction rates and less alteration of the sample appearance.

## Introduction

### State of the art

Archeological wood artifacts are defined as objects carrying traces of cultural activities and giving information about past civilizations [1, 2]. Humans have used wood for millennia for everyday tools and structures. On waterlogged archeological sites, everyday wooden artifacts are commonly recovered [1]. Under specific conditions, the artifacts are very well preserved [2]. This is the case of waterlogged archeological wood (WAW). WAW artifacts are excavated from anoxic environments, such as marine or freshwater [1, 2]. For marine WAW artifacts, the state of degradation may vary depending on the exposure conditions of the artifacts, the wood species, and the duration of burial [1]. In general, the inner parts of the artifacts are more preserved than the outer parts. Outer parts present higher water content, inorganic inclusions, and biopolymer decay [1]. In particular, the cellulose content decreases with degradation, increasing the water content. Moreover, oxygen levels, salinity, or sediment type are the key factors of microbial decay [3]. For example, the *Vasa* and *Mary Rose* warships are two famous WAW marine artifacts with different preservation states. The *Vasa* remained submerged in the harbor of Stockholm for 333 years, which has low salinity and low oxygen concentrations, explaining the preserved state of the ship [2–4]. In contrast, the *Mary Rose* remained submerged and partially buried in the sediment and saline water of the Channel sea for 437 years. Only the parts embedded were recovered as the degradation process was slowed down in those conditions [5, 6]. WAW artifacts are not only buried in marine environments. Lake sites are also waterlogged archeological sites, with wooden embedded artifacts. Many freshwater villages were discovered in Switzerland during the late nineteenth century and early twentieth century [7]. These villages were dated from the Neolithic period (10,000–4500 BC) and Bronze age (3200–600 BC). The poles holding up the dwellings were oftentimes recovered in a comparatively good state of preservation. However, even WAW with a well-preserved structure reveals upon deeper investigations that their surface is soft, and significant alteration occurred within the wood cells and tissue during the burial time [5].

In anoxic environments, as the oxygen content is very low, the main degrading agents of wood are bacteria [2, 3, 8]. The degradation process is slower than in an oxic environment where fungi and other organisms are active. There are two main degrading agents: erosion bacteria (EB)- and sulfate-reducing bacteria (SRB). EB are primary wood degraders in near-anoxic or anoxic conditions [2, 3, 5, 9, 10]. They enhance the degradation process at the surface and work their way inside the wood, breaking down the lignocellulose structure of the cell walls for both soft- and hardwood such as pine and oak [3, 8, 11–13]. EB reach the inner parts through rays and pits [2]. In the secondary cell walls, cellulose is converted into an amorphous substance [5, 13], while the middle lamella, rich in lignin, remains intact [2]. If present, SRB act as secondary wood degraders [13, 14]. SRB use the metabolic products of primary wood degraders as carbon and energy source. They develop in degraded areas, oxidizing the carbohydrates produced as EB degradation products by reduction of sulfate ions (SO_4_^2−^) of the surrounding environment [13–15]. SO_4_^2−^ acts as an electron acceptor, while carbohydrates as an electron donor. Their interaction leads to the formation of gaseous hydrogen sulfide (H_2_S), which accumulates within the degraded cells of the wood. H_2_S can then interact with the lignin content to form organosulfur and/or elemental sulfur. H_2_S can also interact with free Fe^2+^ ions to form iron sulfides within the degraded wood cells [16]. It is worth mentioning that Fe^2+^ ions can act as a catalyst for the degradation of cellulose through the Fenton reaction [12, 13, 17, 18]. In marine and freshwater environments, Fe^2+^ ions are provided by the surrounding environment (*i.e.,* sediments [7]) or corroded iron parts present, as the bolts of the *Vasa* and *Mary Rose* warships [1, 8, 14, 16, 18, 19]. Mackinawite (FeS) and pyrite (FeS_2_) are two of the main iron sulfides observed in WAW [20]. As for marine artifacts, FeS and FeS_2_ are also reported in freshwater WAW, although with lower concentrations.

While stable in anoxic environments, the formed iron sulfides oxidized once are exposed to a different temperature, relative humidity, or oxygen concentration. They convert into iron oxyhydroxides, iron sulfates, elemental sulfur, and/or sulfuric acid. These conversions generate irreversible physical and chemical damages to the wooden artifacts [21]. The precipitated salts increase and occupy more volume, inducing cracks of the structure [13, 17, 21, 22]. The produced acid interacts with the organic matter and hydrolyses the wood. These phenomena result in a loss of strength, structural damage, reduction of mechanical stability, and shrinkage of the structure [13, 17, 22]. In addition, the water evaporation from the wood matrix can lead to the collapse of the already fragile structure, enhancing further structural damage [23]. These phenomena have been observed for both marine and freshwater WAW.

### Current conservation/preservation methods

Since WAW artifacts are carriers of information of past times, their preservation is of great importance [24]. After excavating, WAW exhibits decayed and un-decayed cells. This results in an unstable remaining cell structure that keeps its dimension only because it is either soaked or filled with water. Because the wood cells are weakened by cellulosic degradation, the cells cannot withstand the extremely high surface tension of water and the capillary forces. Thus, the wood's cellular structure collapses when the water and debris bulking the structure are removed during the drying process. The shrinkage is extremely anisotropic so that the shape might be destroyed to such an extent that the object can no longer be identified [24]. Therefore, it is essential to keep the objects in wet, dark, and cold conditions until further treatment [25]. For permanent storage, exhibition, or research, this condition is not reasonable. Thus, a long-term conservation strategy aims to transfer the wooden object into a dry and stable condition, while preserving the dimensions it showed when being excavated. As such, standard conservation methods aim to stabilize the wood cells in order to avoid cell collapse during the necessary drying process. Furthermore, the wooden characteristics, such as color and appearance, ought to be preserved. The method should also be reversible or at least leave the object re-treatable. During the history of WAW conservation, various substances have been used [26–28]. Some of them are still in use or a stage of development; others failed in terms of long-term stability and are no longer in use. One of the most established consolidation agents is polyethylene glycol (PEG), used either by spraying or immersion, often followed by a freeze-drying process. The method is executed with PEG of various molecular sizes, usually chosen according to the following criteria:

- Solid at room temperature
- Not a media for moving ions
- Fast and deep penetration during impregnation
- High eutectic temperature
- Fast and economical freeze-drying

In the last decade, the use of high molecular mass PEGs (HMW PEG) at ranges between 2000 and 4000 as impregnation agents turned into the standard in several institutions in North and Central Europe.

From the middle of the twentieth century, freeze-drying became the most common drying practice to maintain the original volume and surface of waterlogged wood prone to shrink or collapse during the drying process [28]. The principle of vacuum freeze-drying is to decrease the temperature below the freezing point of all fluid components, reduce the air pressure to low pressures and add the required thermal radiation to the system. Under this environment, the drying process takes place through sublimation. The liquid phase is bypassed because capillary forces in liquids can causes collapse in the decayed wooden cells [24]. The benefit of freeze-drying artifacts impregnated with HMW PEGs is their ability to form eutectic mixtures at a defined eutectic temperature (Te) [24, 25]. Keeping product temperatures beneath Te results in dried artifacts with remaining crystalline PEG in order to stabilize the decayed wood structure [24]. The Te of pure HMW PEG water mixtures is sufficiently well-published [25, 29]. For the HMW PEG treatment, special freeze dryers are needed to ensure completely dried and stabilized artifacts through a time-efficient freeze-drying process [24]. It is recommended to keep product temperatures at least 3 °C beneath Te during freeze-drying because the solutions might be affected by contaminations during PEG impregnation [25].

In addition to the consolidation of the structure, the extraction of iron and sulfur is also essential. Iron and sulfur being the main causes of waterlogged wood degradation post-excavation, studies are ongoing to extract them. The methods employed can be applied before or after consolidation and should stabilize the artifacts. Iron is generally chelated by chemical chelating agents, solubilized with acids, or reduced to a more soluble form [30]. Ethylenediaminetetraacetic acid (EDTA) is currently employed as a chelating agent to remove iron stains in the conservation of paper or textile [31]. Indeed, EDTA forms strong complexes with both Fe^2+^ and Fe^3+^ ions [32]. When applied to wood conservation, derivatives of EDTA were investigated, such as ethylenediiminobis (2-hydroxy-4-methyl-phenyl) acetic acid (EDMA), or diethylenetriamine pentaacetic acid (DTPA) [33]. However, these chelating agents are controversial due to their toxicity [30]. Even though the extractions are effective, the complexing agents do not prevent swelling of the wood structure, and the treated pieces presented a reddish hue [33]. The long-term effects of these methods remain unknown. Concerning the neutralization of the sulfuric acid, tests using ammonia gas or immersion in alkaline baths have been run. These treatments are generally applied after consolidation of the wooden artifacts. Immersion in alkaline baths increases the surface's pH, leading to a cellulosic decay of the object [1]. It was also observed that PEG was removed during the immersion time. Treatment with ammonia gas seems more suitable for the object as the pH stabilized in time and the carbohydrates content was less affected [1]. The sulfuric salts are converted into ammonium salts without volume expansion, and thus, no cracks are formed that could endanger the structure. However, the crystallinity of the cellulose decreases with this method, decreasing the artifact's stability. Even after consolidation and extraction, if some Fe/S species remain in the wood matrix, wood degradation will continue through salt precipitation and acidification. Alternatives or optimization of the current treatment is currently investigated [34–38].

### Biotechnologies

In recent years, the interest in more sustainable and eco-friendly solutions has made researchers in conservation look for biological processes that can be used to substitute current treatments. Successful applications of microbial metabolisms for conservation (*i.e.,* bioconservation) are found on numerous different materials [39, 40]. For instance, stone monuments have profited from biomineralization (*i.e.,* the formation of biogenic minerals) treatments, where bacteria induced the precipitation of calcium carbonate. Damaged stone is consolidated by filling the cracks formed due to weathering [41–43]. Metal substrates have also been treated via biomineralization processes. The minerals formed are integrated into the natural corrosion patina creating a compatible passivation layer that is more effective than current coatings [40]. Biopassivation has been studied over more than ten years at the University of Neuchâtel, Switzerland. Specifically, copper-based artifacts have been treated with fungal strains reported to form biogenic oxalates, an ability that allows the transformation of metal compounds into metal oxalates. Metal oxalates, particularly copper oxalates, create attractive compact patinas that are not associated with cyclical corrosion, providing good protection of the surface [44, 45]. In iron objects, biomineralization enhances the chances of survival of the object when the corrosion crust around the object is contaminated with chloride [46]. Iron-reducing bacteria were used under anoxic environments to produce stable biogenic iron minerals, such as vivianite [47, 48]. This precipitate increases the stability of the iron artifacts (biostabilization). Alternatively, biocleaning, focusing on the removal of the reactive chlorides-containing corrosion products, was also explored. This extraction process used alkaliphile fungal strains that accumulate chlorinated species as a detoxification strategy for survival in extreme environments. Promising results showed the removal of the harmful corrosion layer without altering the metal substrate underneath [46].

Some recent research in bioconservation has also focused on WAW. For example, the possibility to use bacteria for the direct consolidation of wood has been investigated [35]. Promising results showed that the treated wood was reinforced thanks to the accumulation of bacterial cellulose [35]. Still, not many studies have focused on the capabilities of microorganisms to preserve WAW. The removal of iron and sulfur species using a biological approach has only been mentioned in few studies [12, 49]. However, the different metabolisms of some microorganisms could be further exploited for the conservation of WAW. Oxidation of iron sulfides by sulfur-oxidizing bacteria is a selective process already established in “biomining” [50]. Some different types of chemolithotrophic bacteria, like *Thiobacillus denitrificans*, are able to grow using sulfur and iron compounds as an energy source and/or reducing agent, at neutral pH [51, 52]. Moreover, *T. denitrificans* is an autotrophic bacterium [51]. Carbon dioxide would be an essential nutrient, but it is not expected for this bacterium to attack the wood. Indeed, *T. denitrificans* have already been proposed to remove iron sulfides from archeological wood due to their specific metabolism [49]. However, the iron contained in the mineral was not considered. Iron precipitates lead to further issues for the preservation of WAW. A possible solution is the use of microbial iron chelators, *i.e.,* siderophores, that will form water-soluble complexes with Fe^3+^. Siderophores are iron-binding compounds with high solubility and low molecular weight, produced by many bacteria under iron starvation. They present a high affinity for Fe^3+^, and they have already been tested in paper and wood artifacts [31, 53].

This study aims to compare a defined biobased treatment involving the co-use of siderophores and *T. denitrificans* to current extraction treatments employed in WAW conservation.

## Materials and method

### Wood samples

Two different wood species were selected to evaluate the biobased extraction method: oak (*Quercus* sp.) and pine (*Pinus* sp.) as mainly reported wood species for WAW [33, 54].

Fresh oak and pine were provided by a carpenter (J.F. Liabeuf, Cernier, Switzerland). The archeological wood samples were obtained from poles of Neolithic oak provided by the Archaeological Service of Bern Canton (ADB) and the Swiss National Museum (SNM), as well as boards of pine (ADB only). All samples were cut from the outermost layer of the object, corresponding to the most degraded part. All the samples presented the three cutting sections: transversal (Tv), tangential (Tg), and radial (Rd). For each wood species and type, 10 sets of 19 samples were prepared. For each set, one sample was kept as an untreated reference (Ref). The set distribution is described in Table 1.

**Table 1:** Sample information and distribution based on wood species, wood type, artificial contamination (AC), and provenance (ADB: Archaeological Service of Bern Canton and SNM: Swiss National Museum)

Set name	Wood species	Wood type	AC	Wood origin
*B*	Oak	Fresh	Yes	Carpenter
*C*	Oak	Archeological	Yes	ADB
*D1*	Oak	Archeological	No	ADB
*D2*	Oak	Archeological	No	SNM
*E*	Pine	Fresh	Yes	Carpenter
*F*	Pine	Archeological	Yes	ADB
*G1*	Pine	Archeological	No	ADB
*G2*	Oak	Archeological	No	SNM

### Mock-ups preparation

Sets B, C, E, and F were artificially contaminated (AC) with the protocol described in Albelda-Berenguer et al. [14]. The samples were first immersed under vacuum (−600 mbars) in a solution of FeCl_2_·4H_2_O 0.5 M for 4 h, oven-dried overnight at 50 °C, then immersed under vacuum (−600 mbars) in a solution of Na_2_S·9H_2_O 0.5 M for 4 h. For sets C and F (archeological samples), no drying overnight was applied to avoid an eventual collapse of these fragile samples. Fresh wood samples (sets B and E) were previously immersed in deionized water for a week to enhance wood degradation [55].

### Extraction methods

Two extraction methods were investigated: an innovative biological extraction and a commonly employed chemical extraction method. Before extraction, the samples were put in 400 ml of deionized water and sonicated for 2 min and then 4 min. The water was changed between the two-sonication baths. The sonication allows removing the chemicals employed for the artificial contamination described above. Six samples per set were used for each extraction method tested and labeled as biologically treated (BT) and chemically treated (CT) samples, respectively. In parallel, six samples remained in deionized water as control and labeled as non-treated (NT) samples. The volume of the extraction solution was 200 ml.

For the biological treatment, *T. denitrificans* (DSMZ 12,475) was obtained from the German Collection of Microorganisms and Cell Cultures (DMSZ, Braunschweig, Germany). The strain was cultivated using standard anaerobic techniques at 30 °C in the dark. Deferoxamine (DFO) was purchased as Desferal® (Novartis International AG, Basel, Switzerland). The BT samples were immersed in a solution of DFO 84 mM for 10 days. Samples were kept at room temperature and with orbital agitation (100 rpm). After rinsing with deionized water, the samples were then immersed with *T. denitrificans* for 20 days. Previous experiments were conducted using bacteria only, but it resulted that higher extraction rates were obtained by combining siderophores and bacteria immersion. Therefore, the two-step combination is presented here as biological treatment (BT). Samples inoculated with the bacteria were incubated at 30 °C in darkness without agitation. Chemically treated samples were immersed in sodium persulfate 0.1 M for one day to oxidize sulfur species. After rinsing with deionized water, the samples were then immersed in EDTA 0.125 M for seven days to complex free iron ions. During the whole process, the samples were kept at room temperature without agitation.

At the end of treatment, BT and CT samples were put in degassed deionized water, and several cycles of 2 × 20 min vacuum (−1 bar) followed by 1-h sonication until constant pH was reached and storage solution conductivity was in the range of 10–30 µS/cm^−1^ (as the rinsing water).

### Conservation protocol

Each partner institution (ADB and SNM) treated five sets of samples divided into 15 groups based on the extraction method: ADB-conserved sets D1, E, F, and G1 while SNM-conserved sets B, C, D2, and G2. For each group, 5 out of 6 cubes were treated, while one sample per set was stored in degazed and deionized water as reference sample for the extraction step in case further analyses would be necessary. The consolidation treatment comprised five sequential baths of 350 ml with increasing PEG 2000 concentrations: 8% (PEG #1), 16% (PEG #2), 24% (PEG #3), 32% (PEG #4) and 40% (PEG #5). Compared to other PEG molecular sizes, the diffusion time of PEG 2000 is acceptable. The eutectic temperature is not too low so that the conservation with PEG 2000 by slowly increasing the concentration was chosen. Deionized water was used without the addition of biocide. Each bath lasted one month. All used PEG solutions were kept for further analysis.

Four cubes per group were freeze-dried, and one remained in 40% PEG solution as a consolidation reference. One sample of the groups C (NT, BT, CT) and B (BT) was equipped with a thermal couple while freeze-drying to monitor the product temperatures. In addition, the weight of two samples (D1 and G2) was continuously measured during the drying process with a spring scale. The procedure lasted 10 days at both institutions with the following settings: vacuum: 0.12 mbar and -50 °C at the condenser (ADB) and 0.12 mbar, and −85 °C at the condenser (SNM), and the product temperature was kept beneath −22 °C. As soon as the weight of samples D1 and G2 stabilized, the temperature of the chamber was gradually raised until room temperature. After the freeze-drying was completed, all cubes were mechanically cleaned with soft brushes. Scalpel or needles and the help of a blow-dryer were necessary to remove excess PEG from the surfaces.

### Analytical protocol

All the characterization methods described below were applied on reference samples (Ref), before (t0), and after (t1) extraction as well as after conservation protocol (t2).

#### Colorimetry

A Minolta CM-508D spectrophotometer was used for the color measurements, with the following parameters: Specular Component Included (SCI), Illuminant D65 (daylight containing UV component, color T 6504 K), d/8° geometry, 10° observer, measurement area diameter 10 mm, illumination with Xe flash light source 100% UV containing all UV components or 0% UV containing no UV components, CIELab 1976 color space.

All the cubes of the sets were characterized. For each cube, one measurement at the center of each face was carried out through a plastic film. The color variation was calculated according to the equation $\Delta E^{*} = \sqrt {\left( {\Delta L^{*} } \right)^{2} + \left( {\Delta a^{*} } \right)^{2} + \left( {\Delta b^{*} } \right)^{2} }$, compared with the reference sample (Ref) of each set.

#### ATR-FTIR spectroscopy

Wood degradation was investigated by Fourier transform infrared spectroscopy. Wood samples were characterized in the 4000–650 cm^−1^ range using a Nicolet iS5 instrument (Thermo Fisher Scientific, Waltham, MA, USA) equipped with an Attenuated Total Reflectance accessory with 16 scans, resolution of 4 cm^−1^, and an analyzed area of 62.5 µm. Two samples per treatment (BT, CT, and NT) and per set were analyzed, and three spectra per five faces were recorded for a total of 90 spectra per set.

Three ratios were calculated based on the height of vibrational bands at 1034 cm^−1^ assigned to holocellulose H and lignin L, 1158, 1374 cm^−1^ assigned to H, cellulose C and 1506 cm^−1^ assigned to L: R1 = I(1158)/I(1506), R2 = I(1374)/I(1506) and R3 = I(1034)/I(1506) [56, 57]. These ratios permit determining whether the wood components were affected by the treatments and thus the wood state of degradation. After correction with a polynomial function and the normalization of the spectra, the heights of the selected bands were measured using Rstudio v3.6.2 software (Rstudio, Boston, MA, USA) and ChemoSpec package (Bryan A. Hanson, DePauw University, https: //bryanhanson.github.io/ChemoSpec/index.html).

#### Raman spectroscopy

Compounds present at the surface of the wood samples were identified by Raman spectroscopy. For wet samples (before conservation protocols), the measurements were taken on the samples immersed in their stock solution (degassed water). This methodology allows avoiding over warming of the samples that could induce a possible modification of the compounds under the laser excitation. For dry samples (after conservation protocols), the measurements were taken directly on the wood faces, without preparation of the samples. Analyses were carried out using a LabRam Aramis Horiba instrument (HORIBA Jobin Yvon GmbH, Bensheim, Germany) and LabSpec 5 software with a 632.8 nm laser, in the range 100–1500 cm^−1^, with 100 × objective (numerical aperture of 0.9) and laser power of 0.99 mW at the surface of the sample (D1 filter, 10%), 1800 lines/mm grating, 1000-µm confocal pinhole and 100-µm spectrometer entrance slit. Two samples per treatment (BT, CT, and NT) and per set were analyzed. One spectrum at the center of three faces corresponding to the three cutting sections (Tv, Tg, and Rd) was recorded: two spectra as routine ones (10 acquisitions of 5 secs) and another as a long acquisition (25 acquisitions × 10 s). A total of 18 spectra per set were recorded. Moreover, water pre-immersed wood and archeological samples of different wood types were analyzed to obtain a reference spectrum of the wood substrates.

#### Inductively coupled plasma–optical emission spectroscopy (ICP–OES)

The quantification of iron and sulfur species was done using ICP–OES. An Optima 2100 PerkinElmer (PerkinElmer, Waltham, MA, USA) was employed to determine the species concentrations. The analyses were performed on digested wood samples and solutions after extraction and consolidation. One treated wood sample per set and treatment was analyzed after digestion in nitric acid HNO_3_ 65% ultrapure (Suprapur, Merck KGaA) by reflux for 24 h. The extraction rates were calculated based on the species remaining in the wood samples before and after extraction.

### Statistic approach

Chemometric analyses were performed as a validation method to investigate the efficiency of the artificial contamination before extraction treatments were applied. The characteristics of BT and CT were also evaluated in terms of extraction efficiency, visual appearance, and innocuousness with respect to the already weak wood structure. Principal component analyses (PCA) were carried out on all spectroscopic measurements. Additional analyses of variance (ANOVA) were performed on ATR-FTIR ratios, color coordinates, and iron sulfide extraction. Rstudio software was used. The spectra dataset was corrected (baseline, bin, normalization, Savitzky–Golay filter) before any analyses.

## Results

Results of sets B (fresh oak), D1 (archaeological oak), E (fresh pine), and G1 (archeological pine) will be presented and discussed as representative of this study.

### Colorimetry

**Fig. 1:**
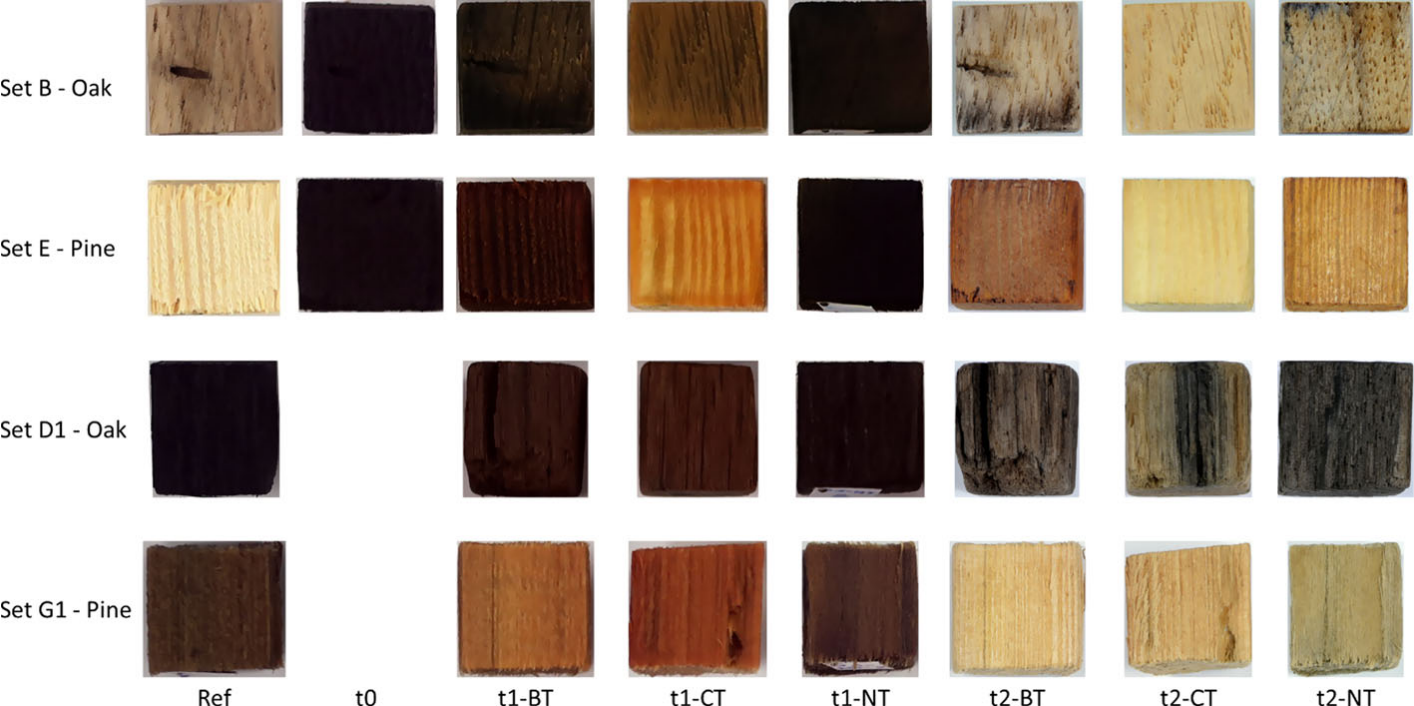
Visual appearance of fresh and archeological oak (sets B and D1) and pine (sets E and G1) samples before extraction (Ref & t0) and after extraction (t1) and after conservation protocol (t2)

Reference samples (Fig. 1 Ref) showed differences between fresh and archeological wood. Archeological wood samples (sets D1 and G1) were darker than fresh wood samples (sets B and E). Submitting these sets (B and E) to artificial contamination allowed to obtain mock-up samples with a similar dark hue as archeological wood samples (sets D1 and G1). After extraction (t1), differences regarding the visual appearance of the samples were observed (Fig. 1 t1). CT samples presented high bleaching compared to samples treated with BT, except G1 set where CT and BT samples are similar appearances. NT samples remained with a dark appearance, as before extraction (Ref and t0). After conservation, which comprised PEG consolidation and freeze-drying (Fig. 1 t2), all sets presented visual alteration that tended to a lighter coloration of the wood with respect to t0 and t1.

Color variations (ΔE*) were performed to ascertain the visual observations (Table 2). Before extraction (t0), artificially contaminated sets (B/E) presented high ΔE*, confirming that the artificial contamination protocol allowed to simulate the appearance of waterlogged archeological wood. After extraction (t1), differences can be observed depending on the treatment. NT and BT samples remained with similar colorimetric coordinates, as their ΔE* slightly varied for fresh samples (sets B/E). Important color variations were observed for D1-CT, G1-BT, and G1-CT. After conservation (t2), color variation values decreased for fresh sets (B/E) while increased for archeological sets (D1/G1).

**Table 2:** Color variations (ΔE*) compared to reference sample (Ref) for fresh and archeological oak (sets B and D1) and pine (sets E and G1) samples before (t0) and after (t1) extraction and after conservation protocols (t2) for biologically (BT) and chemically (CT) treated samples, compared with untreated (NT) samples. Standard error indicated in bracket

Set	ΔE* t0			ΔE* t1			ΔE* t2		
	*BT*	*CT*	*NT*	*BT*	*CT*	*NT*	*BT*	*CT*	*NT*
*B*	47.1 (± 0.8)	46.9 (± 0.4)	46.7 (± 0.8)	41.8 (± 1.3)	24.3 (± 4.3)	54.9 (± 1.7)	17.5 (± 3.1)	6.5 (± 2.3)	23.4 (± 2.4)
*D1*	0.0 (± 0)	0.0 (± 0)	0.0 (± 0)	5.1 (± 1.9)	13.7 (± 1.1)	1.3 (± 0.9)	15.7 (± 0.6)	20.8 (± 1.6)	12.5 (± 0.3)
*E*	57.5 (± 1.2)	58.2 (± 0.9)	57.7 (± 0.5)	51.5 (± 0.5)	26.8 (± 3.4)	50.1 (± 4.5)	28.7 (± 1.8)	14.1 (± 0.7)	24.3 (± 1.4)
*G1*	0.0 (± 0)	0.0 (± 0)	0.0 (± 0)	14.9 (± 1.6)	13.5 (± 1.5)	3.5 (± 2.0)	26.3 (± 0.4)	22.4 (± 1.2)	17.7 (± 5.8)

### ATR-FTIR spectroscopy

**Fig. 2:**
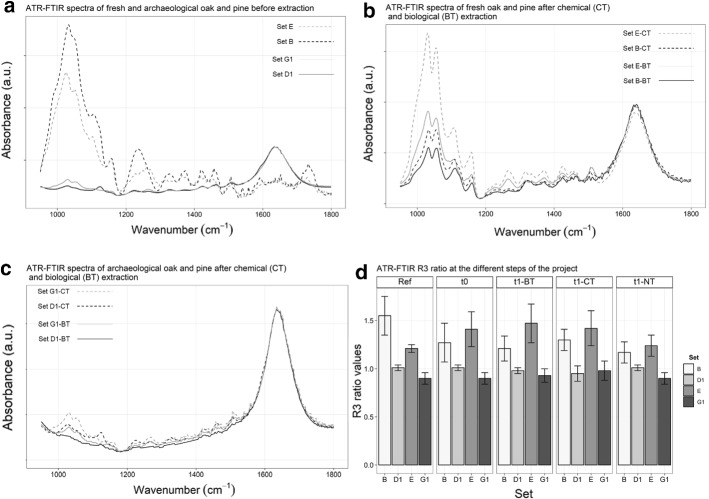
Representative ATR-FTIR spectroscopy spectra in the 950–1800 cm^−1^ region. **a** Before extraction for archeological (–-) and fresh (–-) oak (black) and pine (gray), after extraction for **b** fresh oak and **c** archeological oak (black) and pine (gray) depending on BT (–-) and CT (–-). **d** ATR-FTIR R3 ratios after extraction, depending on steps, extraction method, and set

The degree of wood degradation was evaluated by ATR-FTIR spectroscopy. This approach allowed to investigate the innocuousness of BT and CT for wood material. Recovered and uncontaminated samples (Ref) were considered as starting point. Differences could be observed before extraction between spectra from fresh and archeological wood. The holocellulose bands (1034 and 1059 cm^−1^) were intense for fresh wood, while their intensities decreased for archeological wood (Fig. 2a). After both extraction methods, the spectra from BT and CT samples were different from NT ones. Even though the bands of fresh wood were still more intense than archeological wood, the band at 1738 cm^−1^ assigned to xylan content (a component of hemicellulose) [18, 58] was no longer visible for any set (Fig. 2b and c). Also, the band for water adsorption was then visible for all sets, at 1650 cm^−1^.

ATR-FTIR ratios showed similar values before and after extraction (Fig. 2D, t0 & t1). Ratios R1 and R2 (holocellulose/lignin and cellulose/lignin, respectively) presented similar values, whatever the wood species or the extraction treatment (data not shown). With ratio R3 (holocellulose/lignin), differences could be observed between fresh and archeological woods.

After conservation, mainly PEG was observed with ATR-FTIR spectroscopy. The band at 1506 cm^−1^, assigned to lignin, was still observed but all the ones in the range 1000–1400 cm^−1^, assigned to carbohydrates content [18, 58, 59], overlapped with the one of PEG [60].

### Raman spectroscopy

Before extraction (t0), reduced sulfur compounds were identified on artificially contaminated samples. Except for set B, partially oxidized mackinawite (Fe_1-x_S) was mainly identified for all sets. Elemental sulfur (α-S_8_) was identified on set B and NT samples of set E (Table 3 t0). After extraction with BT, only bands assigned to the wood were observed with Raman spectroscopy [61]. On the contrary, set E-CT and E-NT samples still presented reduced sulfur compounds (Table 3 t1). For NT samples, Fe_1-x_S and α-S_8_ were identified in the same proportion as at t0. Concerning archeological wood (sets D1 and G1), no reduced sulfur compounds were identified neither before nor after extraction. The bands observed were identified as wood components. After conservation, PEG was identified on most of the samples. In addition, α-S_8_ was still present on set E-NT and E-BT samples; however, its occurrence is slightly lower than at t1 (Table 3 t2). The characteristic bands at 252, 379 cm^−1^ of lepidocrocite, γ-FeOOH, were also identified on set E-BT samples (medium occurrence) [62].

**Table 3:** Occurrence (•: low; ••: medium; •••: high) of the compounds (M: mackinawite; S: sulfur; PEG: polyethylene glycol; L: lepidocrocite) identified by Raman spectroscopy for each set and treatment (NT: untreated, BT: biologically treated, CT: chemically treated)

Set		t0-Before extraction	t1-After extraction	t2-After consolidation
*B*	*NT*	S (••)	S (•••)	PEG (••)
	*BT*	S (•••)		
	*CT*	S (•••)		
*D1*	*NT*			
	*BT*			
	*CT*			PEG (•)
*E*	*NT*	M (••), S (••)	M (•), S (••)	S (•), PEG (•)
	*BT*	M (•••)		S (•), PEG (••), L (••)
	*CT*	M (•••)	S (••)	PEG (••)
*G1*	*NT*			PEG (•)
	*BT*			PEG (•)
	*CT*			

### Efficiency of extraction

The efficiency of the extraction procedures was evaluated by ICP-OES. From ICP-OES measurements, the extraction rates of iron Fe and sulfur S were calculated. Concerning iron, high extraction rates were obtained for artificially contaminated samples (B/E) but very low for archeological samples (D1/G1) (Table 4) with CT. In contrast, medium-to-high extraction rates were found with BT, independent of natural or artificial contamination.

**Table 4:** ICP-OES iron (Fe) and sulfur (S) extraction rate for biological (BT) and chemical (CT) extraction methods

Set	Fe extraction rate (%)		S extraction rate (%)	
	*BT*	*CT*	*BT*	*CT*
*B*	72.9 (± 6.1)	62.9 (± 8.4)	41.51 (± 4.3)	0.0 (± 0)
*D1*	65.1 (± 4.8)	6.6 (± 12.9)	32.1 (± 5.3)	0.0 (± 0)
*E*	64.1 (± 5.3)	99.8 (± 0)	44.2 (± 6.1)	40.6 (± 6.5)
*G1*	46.2 (± 2.2)	0.0 (± 0)	0.0 (± 0)	7.3 (± 3.1)

Concerning sulfur S, extracted S was detected only for pine samples treated with CT (E/G1), while BT extracted S for all sets, except archeological pine (set G1).

ICP-OES analyses performed on the solutions after extraction showed the presence of both iron and sulfur species (data not shown). Yet, the sum of species concentration in the extracted samples and from the extraction solutions are not equal to the concentration of the species of untreated samples.

### Consolidation treatment

After PEG baths #3 (24%) and #4 (32%), two samples from set B-NT and B-BT began to float beneath the water surface. No further PEG could penetrate the wood, and the difference in density resulted in an increased lightness of the samples. The weight also dropped slightly. All other samples remained at the bottom of their PEG bath until the end of the consolidation treatment.

Iron and sulfur species were also detected within consolidation solutions. Their concentrations decrease with the renewal of the consolidation baths.

### Chemometrics

Chemometrics were applied to the spectroscopic data to ascertain the previous observations. First, PCAs were performed on the ATR-FTIR and Raman spectra at the three phases of the project (t0, t1, t2).

**Fig. 3:**
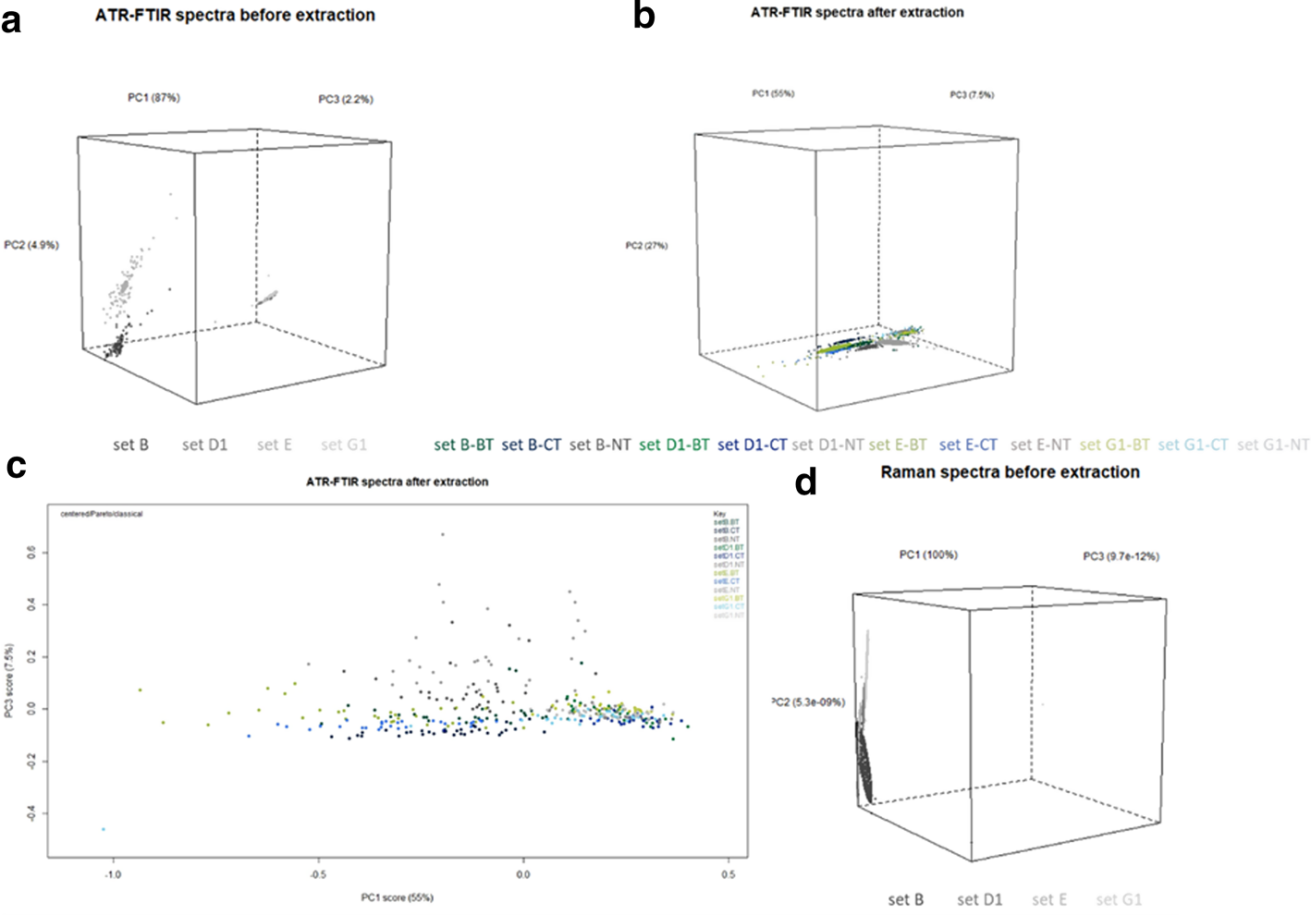
3D score plot PCA on ATR-FTIR spectra **a** before and **b** after extraction. **c** PCA on PC1 and PC3 for ATR-FTIR spectra after extraction. **d** 3D score plot PCA on Raman spectra before extraction

Concerning ATR-FTIR, PCA was able to cluster the different samples based on their type (fresh or archeological) before extraction (t0) (Fig. 3a). Though, after extraction (t1), no distinct clusters were visible among the samples (Fig. 3b and c). Yet, fresh samples (sets B and E) gathered in negative parts of PC1 influenced by holocellulose bands. PC3 showed B-NT and E-NT, which gathered in positive parts. Lignin vibrational bands affected negatively PC3, suggesting that lignin bands were less intense for B-NT and E-NT. At t2, PEG was mainly identified on the samples, explaining that no cluster formed. Concerning the Raman data, PCA did not allow the samples to be clustered at t0 (Fig. 3d). Yet, differences between fresh and archeological and treated or not samples were still visible on ATR-FTIR and Raman spectra.

Analysis of variance (ANOVA) was then performed on the ATR-FTIR and ICP-OES results with samples before extraction as reference. Results on oak wood are presented in Table 5. ATR-FTIR R1 and R3 ratios showed a significant difference when the samples were artificially contaminated or not for the type of wood (fresh *vs.* archeological) and the wood species. On the contrary, BT and CT did not show significant differences among them but with untreated (NT) samples. The interactions among the predictors are described in Table 5.

**Table 5:** ANOVA for oak wood depending on predictors (artificially contaminated: AC; fresh or archeological wood: Type; BT, CT and NT method: Treatment)

Predictors	ATR-FTIR R1/R3 ratios	Iron extraction	Sulfur extraction
*AC*	***	***	
*Type*	***	***	
*Treatment*	**	*	*
*AC:Type*	***		
*AC:Treatment*	*	***	***
*Type:Treatment*	***	***	***
*AC:Type:Treatment*			

*p*-val < 0.05 ‘*’,  *p*-val <  0.01 ‘**’,  *p*-val < 0.0001 ‘***’

The effect of predictors showed that AC:Treatment and Type:Treatment gave significant differences compared to the ATR-FTIR ratios before extraction. Concerning the ATR-FTIR R2 ratio, no significant differences were observed.

ANOVAs on the iron and sulfur extraction efficiencies revealed the extraction method gave significantly different results as well as the effect of predictors AC:Treatment and Type:Treatment.

## Discussion

### Efficiency of the extraction

#### Sulfur extraction

After extraction, Raman analyses did not detect reduced sulfur compounds on the surface of BT samples, while elemental sulfur was still identified on set B-CT samples. Average sulfur extraction for half of the sets was around 40% for both extraction methods, while the other sets presented no extraction (G1-BT, B-CT, and D1-CT) or very low extraction (set G1-CT) (Table 4). BT seemed to be more efficient to extract sulfur species, with higher S extraction rates for artificially contaminated sets (B/E). The extraction rates were generally higher for oak wood species. ANOVAs validated these observations with a p-value < 0.05 regarding the predictor treatment and wood species. The wood type did not show significant variation for oak species, confirming the efficiency of BT for both fresh and archeological wood. Regarding pinewood, the wood type affected the ANOVA. Both D1 and G1 sets are archeological samples used as a reference for natural iron and sulfur contamination. Their contamination with iron and sulfur here could be heterogeneous and in fewer amounts than artificial contamination. Indeed, Raman analysis already skipped the detection of reduced sulfur before extraction. For the fresh samples, the contamination was homogeneous, with all the samples being contaminated with iron and sulfur species. As extraction rates for sulfur were around 40%, the extraction may have been more efficient on the surface, explaining the results obtained with Raman. Concerning CT, sulfur extraction seemed more efficient on pinewood. ANOVA showed a difference between the extraction method, with a *p*-val > 0.05, as well as the effect of the wood type (fresh *vs.* archeological) and treatment (Table 5). Sodium persulfate was used as a pre-extraction treatment to oxidize the iron sulfide compounds, allowing the highest extraction of sulfur species, as observed during a French study [63]. However, the occurrence of sulfur at the surface of CT samples was lower than the proportion of FeS*x* and α-S_8_ identified before extraction (Table 3). All the sulfide ions were not extracted as expected by sodium persulfate immersion treatment. However, a tendency could be observed for both extraction methods. As discussed above, BT was more efficient regarding oak wood while CT for pinewood. The wood species may then influence the efficiency of the extraction method employed.

#### Iron extraction

No iron compounds were detected after extraction by Raman spectroscopy. Yet, as for sulfur extraction, BT extraction rates of iron were not 100%. The extraction of iron must have also been more efficient on the uppermost layer. Current methods applied in the conservation of wood that uses strong iron chelators show iron extraction rates that vary from 60 to 100% [33, 63], as confirmed by extraction rates obtained with CT for artificially contaminated samples (sets B/E) (Table 4). BT iron extraction rates are within the range of current chemical extraction methods. However, these studies were primarily focused on objects that presented already oxidized forms of iron sulfides or different wood species, both factors that could influence the extraction procedure [33, 63]. Application of CT confirmed that high iron extraction rates are obtained for iron sulfide compounds for both hard- and softwood artificially contaminated. Especially for set E, the extraction rate was close to 100%, suggesting an extraction not only at the surface but also in-depth.

PCA was performed on the Raman spectroscopic database. The analysis did not reveal any information about the data. All the spectra clustered, independently of the compounds identified before and after extraction and independently of the extraction treatment applied. No prediction could be obtained from the data. Further investigations were then performed. For artificially contaminated samples, ANOVAs on the ATR-FTIR and ICP-OES results showed significant extraction rates for BT and CT and hard- and softwood species. Applied on fresh wood, BT and CT led to similar extraction results. For archeological wood, the wood species influenced the iron extraction efficiency. Oak presented a higher extraction rate, but also the treatment, as BT is significantly different to CT only for archeological pine (set G1) (*p*-val > 0.05).

Different factors influence the extraction rate: (1) diffusion of the ligand into the matrix, (2) formation of the iron–ligand complex, (3) diffusion of the complex out from the wood, and (4) amount of iron–chelator complex already existing in solution [64]. No further experiments were carried out at this point to define formation constants or diffusion rates. However, we observed that even after intensive rinsing of the cubes with deionized water, the iron–DFO complex may be released from the BT cubes. A slow diffusion of the complex out of the wood cubes was assumed. Nonetheless, similar iron extraction rates in the majority of the sets treated with BT give encouraging results in terms of application of DFO to different wooden objects with different characteristics (*i.e.,* degradation state, wood species, mineralogical composition of iron and sulfur compounds, etc.). The extraction rates can be increased by an increased application time or renewal of the chelating solution [64].

Iron sulfides such as mackinawite (FeS), with a relation 1:1, could translate to similar extraction rates for iron and sulfur. For these compounds, a correlation exists between the extraction of iron and sulfur. In general, when the extraction of iron is higher, sulfur extraction is also increased [33]. In our samples, 1:1 correlation appeared for samples of set E-BT. The sets with higher iron extraction than sulfur (B, D1, and G1) may have presented other iron and/or sulfur species apart from iron sulfides, like iron oxides or organosulfur.

### Visual appearance

The visual appearance of the samples was correlated with the iron and sulfur extraction. While very dark before extraction, G1-BT, E-CT, and G1-CT samples presented bleaching (Fig. 1). All these sets are softwood, suggesting that softwood species would be more sensitive to extraction treatments. ANOVAs showed that for archeological pinewood especially, application of BT and CT led to significant variations regarding the color variation (*p*-val < 0.05). Past studies have used recovering the original color as an indicator for successful iron extraction [63]. The iron extraction rates for BT were 74.9 and 64.1% for fresh oak and pine, respectively. The BT samples maintained a closer aesthetical appearance to samples after artificial contamination, although a considerable amount of iron was extracted. For archeological wood sets, ΔE* was lower, but some variations were still observed, with ΔE* values over 5. ΔE* = 2.3 is the threshold where the color variation is visible by the human eye [65]. CT samples were modified, with pinewood sets being more altered (*p*-val < 0.05). However, comparing with Ref samples, artificially contaminated samples treated with CT displayed lower color variation ΔE* than samples treated with BT. Then, for fresh wood, CT would allow recovering a visual appearance closer to the original one, *i.e.,* Ref samples (Fig. 1). Archeological sets also presented an important ΔE* with oak wood being more affected than pinewood, based on the color variations measurements. Set G1 displayed a red appearance for both BT and CT. Concerning set G1, both extraction methods were too harsh and did not respect the recovery of the original surface. However, ANOVA did not reveal significant differences among the two wood species. The utilization of sodium persulfate during CT may be the reason for extensive samples' bleaching. Persulfate ions are employed in several fields as a bleaching agent [66, 67]. But this discoloration could also be attributed to the extraction of iron ions. Indeed, iron stains paper and wood in a very typical way [63]. When present, iron oxides made reddish spots on the substrate, as observed here for NT samples (Fig. 1). However, set G1 (archeological pine) presented a red surface, previously observed on some waterlogged wood artifacts treated with strong complexing agents [33]. The discoloration suggested the extraction of the salts within the wood but also a transformation of the wooden structure.

After conservation protocols, BT and CT samples' appearance was altered. Conservation treatments usually result in a modification of the hue [68]. For fresh oak, the original surface (Ref) was almost recovered with BT and CT with an ΔE* value for B-CT close to 5 as if no treatment was applied to the samples. Set E treated with BT presented a reddish hue. Raman spectroscopy revealed the presence of lepidocrocite, an iron oxide, at the surface of E-BT samples. It is possible that BT was effective superficially, and the remaining iron in the core diffused toward the surface over time. Also, the iron-DFO complex has a strong red coloration [31]. As discussed above, the diffusion of the complex out of the wood was slow, and further analyses are needed to determine whether all the complex was adequately washed out of the wood cubes. E-CT also displayed a very bright appearance with its ΔE* value above 5. All the other sets treated with CT showed a very bright appearance similar to the fresh wood. CT is probably then a too strong chelating agent as archeological samples were as bleached as fresh wood. For these samples, the final coloration does not respect conservation criteria. Indeed, the original surface aspect of the samples after recovery should be obtained also after treatment, which is not the case here for G1-CT samples, though ANOVAs did not show significant variation for all the predictors. During the consolidation with PEG, some iron ions present in wood depth may have been released as some baths still presented a strong orange to brown discoloration. In general, PEG baths of artificially contaminated samples treated with BT displayed such coloration. The species may have finally reached the surface and oxidized during the consolidation process. The coloration of consolidation baths for BT could also be explained by the washing out of DFO. It is worth mentioning that the only immersion in PEG consolidation baths could seem to be sufficient to extract the harmful Fe/S species from NT samples. Yet, the release of iron species within the PEG baths may come from the instability of the compounds presented within the samples. Their final appearance was similar to BT and CT samples, and some of the PEG baths also presented high discoloration. First analyses of PEG #1 revealed the presence of iron species, with 40 mg of iron per liter for artificially contaminated sets (B/E). The concentration decreased with the renewal of PEG baths. However, this could be harmful for the artifact's integrity as some studies demonstrated that PEG could act as an electrolyte for iron ions and thus induce an oxidation of the polymer as well as cellulosic degradation [69–71]. Raman investigations revealed the presence of sulfur and lepidocrocite for sets E-NT and F-NT, respectively, after conservation (t2). If PEG could be an extraction method, it did not prevent the oxidation and precipitation of harmful minerals. So, the presence of iron species should be avoided as much as possible before the consolidation step starts. Further investigations of iron, sulfur, and iron-chelating agents will be performed to identify which compounds were released during consolidation.

### Risks’ evaluation

The innocuousness of each extraction method regarding wood substrate was evaluated through ATR-FTIR spectroscopy, as widely reported in WAW literature [72, 73]. Whatever the extraction method employed, the ATR-FTIR ratios remained in the same range for all sets, and no differences were observed on the ATR-FTIR spectra (Fig. 2b, c, and d). Neither BT nor CT induced further degradation to the wood samples. They seemed both harmless when applied on fresh and archeological wood in line with conservation ethics [78] and promising results regarding BT. This observation is encouraging regarding the application of BT on WAW artifacts. However, the holocellulose content seemed to impact less the classification of the spectra as PC1 displayed only 55% of total variance after extraction, compared to the 87% before extraction. PC1 loading presented holocellulose bands in the negative scores. PC3, presenting only 7.5% of the total variance, seemed to influence also the clustering of the spectra. Loading of this PC was positive for bands of aromatic skeleton and CO groups of lignin, in general (1219, 1252, 1267, 1422, 1506, 1583 cm^−1^) [18, 58, 74]. Another plot with only PC1 and PC3 involved was then achieved. The 2D score plot (Fig. 3c) did show that archeological samples still gathered in the positive scores of PC1 while fresh sets were in the negative scores. The overlapping is more important than at t0 (before extraction), but a difference based on the holocellulose bands is still possible. Some samples showed scores in the positives part of PC3, *i.e.,* lignin content. These samples are from fresh sets (B/E), though another internal parameter influences the clustering of the data.

Further investigations of the loadings and ATR-FTIR bands should be done to understand the classification of ATR-FTIR spectra. Compared with ratios before extraction, ANOVA showed significant differences for fresh sets (B/E). But all the ratios obtained after extraction were significantly similar, according to ANOVA. BT and CT were then harmless regarding further wood degradation. However, even NT ratios displayed similar values than BT and CT after extraction. The non-treatment of the wood samples did not induce further degradation, as expected, and was previously observed on genuine WAW artifacts [21], though this study was performed over a short period of time that may not represent the oxidation rates of iron sulfides within WAW. Concerning archeological wood, the ratios also remained in the same range except for set D1-BT. This ratio was significantly different from the one before extraction. On real objects, the wood species may influence the efficiency of the extraction as the stability of the mechanical strength. Cellulose content was untouched during extraction as observed by ANOVAs of ATR-FTIR ratio R2 (Table 5). It can then be suggested that the extraction slightly impacted the holocellulose content and weaken the wood structure. To ascertain this hypothesis, the holocellulose content can be evaluated by chlorination of wood materials [79].

At t2 (after conservation protocols), we could assume that if some iron species remained in the samples and oxidized as demonstrated by Raman spectroscopy, the wood structure could be damaged and lose strength leading to a collapse of the structure [21]. Iron can interact with PEG and then induce even more degradation of both PEG and cellulose. In this case, the integrity of the object will not be preserved. To ascertain this, microscopic analyses of the samples at the different steps of the study will be performed. With scanning electron microscopy (SEM), it will be possible to obtain clear pictures of the wood cells at t0, t1, and t2 and evaluate whether BT decayed the cells, CT, NT, or the conservation protocols. Coupled with maximum water content measurements, the wood degradation grade could then be determined, and the effect of conservation protocols on BT-treated samples evaluated [81]. Additional analyses with energy-dispersive X-ray spectroscopy (EDS) and SEM–EDS will also inform the compound formed or remaining, as complementary analyses to Raman spectroscopy. Shrinkage of the samples after freeze-drying will also be investigated to evaluate the effect of water evaporation on treated samples. The pre-tests showed that the different extraction methods did not have any remarkable impact on the freezing and melting behavior of the tested solutions.

## Conclusions

This study showed the feasibility of an alternative biological extraction of iron and sulfur species from waterlogged archeological wood. Compared to the standard chemical extraction method, biological extraction (BT) was more efficient, at least superficially, as no reduced sulfur compounds were identified by Raman measurements performed on the sample's surfaces. Though ICP–OES measurements showed that the extraction rates were not complete, some iron and sulfur remained inside the samples. Iron was detected in the consolidation bath, especially for BT samples. The iron may be released from the samples themselves or may provide from siderophores chelators (DFO), which were not washed out properly. An optimization of BT should be considered, so all iron and sulfur are extracted during treatment with siderophores and bacteria, not in the following steps. Longer immersion time will be the first parameter to investigate, as BT showed innocuousness regarding the wood structure and visual appearance. All these results were validated by PCA and ANOVAs. To further validate BT as an alternative extraction method, accelerated aging of the samples will be performed according to the ASTM G151 standard. The wood shrinkage and salts precipitation will be investigated to determine whether BT allows a long-term efficiency of the wooden artifacts before being applied on actual waterlogged wood artifacts.

## Data Availability

The datasets generated during and/or analyzed during the current study are available from the corresponding author on reasonable request.
